# A sportomics soccer investigation unveils an exercise-induced shift in tyrosine metabolism leading to hawkinsinuria

**DOI:** 10.3389/fnut.2023.1169188

**Published:** 2023-06-13

**Authors:** Thássia Casado Lima França, Renan Muniz-Santos, Luiz Carlos Caetano, Gustavo H. M. F. Souza, Henrique Fonseca Goulart, Marcio Assis, Altamiro Bottino, Adriana Bassini, Antonio Euzébio Goulart Santana, Eduardo Seixas Prado, L. C. Cameron

**Affiliations:** ^1^Laboratory for Research in Physical Exercise and Metabolism, Federal University of Alagoas, Maceió, Alagoas, Brazil; ^2^Laboratory of Protein Biochemistry, Federal University of State of Rio de Janeiro, Rio de Janeiro, Brazil; ^3^Institute of Chemistry and Biotechnology, Federal University of Alagoas, Maceió, Alagoas, Brazil; ^4^MS Applications and Development Laboratory, Waters Corporation, São Paulo, Brazil; ^5^Research Laboratory on Natural Resources, Federal University of Alagoas, Maceió, Alagoas, Brazil; ^6^Youth Soccer Teams, Fluminense Football Club, Rio de Janeiro, Brazil; ^7^Health and Performance Center/Soccer Professional Team, Sociedade Esportiva Palmeiras, São Paulo, Brazil

**Keywords:** neurometabolism, amino acids metabolism, exercise metabolome, metabolomics, biochemistry of exercise, metabolic pathways, sports science

## Abstract

Tyrosine metabolism has an intense role in the synthesis of neurotransmitters. Our study used an untargeted, sportomics-based analysis of urine samples to investigate changes in metabolism during a soccer match in 30 male junior professional soccer players. Samples were collected before and after the match and analyzed using liquid chromatography and mass spectrometry. Results showed significant changes in tyrosine metabolism. Exercise caused a downregulation of the homogentisate metabolites 4-maleylacetoacetate and succinylacetone to 20% (*p* = 4.69E−5) and 16% (*p* = 4.25E−14), respectively. 4-Hydroxyphenylpyruvate, a homogentisate precursor, was found to be upregulated by 26% (*p* = 7.20E−3). The concentration of hawkinsin and its metabolite 4-hydroxycyclohexyl acetate increased ~six-fold (*p* = 1.49E−6 and *p* = 9.81E−6, respectively). Different DOPA metabolism pathways were also affected by exercise. DOPA and dopaquinone increased four-to six-fold (*p* = 5.62E−14 and *p* = 4.98E−13, respectively). 3-Methoxytyrosine, indole-5,6-quinone, and melanin were downregulated from 1 to 25%, as were dopamine and tyramine (decreasing to up to 5% or 80%; *p*= 5.62E−14 and *p* = 2.47E−2, respectively). Blood TCO_2_ decreased as well as urinary glutathione and glutamate (40% and 10% respectively) associated with a two-fold increase in pyroglutamate. Our study found unexpected similarities between exercise-induced changes in metabolism and the inherited disorder Hawkinsinuria, suggesting a possible transient condition called exercise-induced hawkinsinuria (EIh). Additionally, our research suggests changes in DOPA pathways may be involved. Our findings suggest that soccer exercise could be used as a model to search for potential countermeasures in Hawkinsinuria and other tyrosine metabolism disorders.

## 1. Introduction

Exercise can potentially impact the human metabolome (1, 2). The application of “-Omics” sciences in the field of play of various sports was conceived as sportomics, aiming to investigate the real challenges athletes face, using sports as a model of metabolic stress (3, 4). Sportomics, like all “-Omics” sciences, depends on analytical methods with high processing power to analyze massive volumes of data and is a promising field of investigation (5). Although the number of metabolomic studies in sports remains limited, it continues to grow, supporting investigations in translational sports medicine (3, 6).

We have used sportomics to understand exercise-induced metabolic alterations in sports, including soccer (7). Several recent investigations of amino acid metabolism have focused on the production of ammonia, a toxic metabolite (8, 9). And, we have been studying metabolic changes in response to exercise, especially related to peripheral and central fatigue, such as exercise-induced hyperammonemia (1). There needs to be more information available about the role of amino acid metabolism in biomarker discovery that may help elucidate performance changes during exercise and physiological and physiopathological conditions.

Analysis through a noninvasive sample, such as urine, supports the understanding of the metabolic responses induced by exercise and can lead us to investigate future practical interventions. Under these conditions, we hypothesized that a Sportomics approach might reveal new markers that influence performance during exercise. We previously performed a sportomics investigation of semiprofessional soccer players during a match by mass spectrometry nontargeted analysis (NTA). We were able to identify 3,500+ metabolites in urine. We previously focused on purine metabolism Prado et al. (7). In this study, other data and metabolic pathways were reanalyzed. Through our data, we have identified an exercise-induced metabolic deviation in tyrosine metabolism, resulting in various changes. In this sense, we evaluate these metabolic alterations, pinpointing potential key analytes that warrant further investigation to enhance our understanding of tyrosine metabolism during metabolic challenges.

## 2. Materials and methods

### 2.1. Participants

Thirty male soccer players (19.2 ± 0.2 years old; 71.5 ± 2.2 kg; 1.78 ± 0.01 m) from a junior professional team, playing in the main soccer national league and affiliated with the Confederação Brasileira de Futebol (CBF, Brazilian Soccer Confederation), participated in this study as volunteers. The players were healthy and did not have detectable diseases. The athletes underwent routine medical evaluations, including bimonthly clinical exams and regular assessments of general physiological parameters twice a week. Pre-participation exams were also conducted regularly by the medical department of the soccer club. Participants were instructed not to ingest any supplement or medication in the 10 days prior to the experiment. The athletes were in the training ground canteen, during which their dietary habits were carefully monitored and controlled. Overall, the athletes were following a similar diet, although there may have been some individual variations in taste preferences. The participants were informed previously about the study, and written informed consent was obtained from each subject. All procedures were performed according to the ethical standards of the Ethics Committee for Human Research at the Federal University of the State of Rio de Janeiro (117/2007, renewed in 2011) and met the requirements for regulating research on human participants (Health National Council, Brazil, 1996). The participants were tested during the same match (*n* = 30).

### 2.2. Experimental design, sample collection, and preparation

Two different urinary samples were collected, one immediately prior to and another one immediately after a soccer match (PRE vs. POST).

Urine samples were immediately transferred to a dry ice cooler and transported to the laboratory. Later, the samples were stored in an ultralow temperature freezer (−80°C) until they were prepared for liquid chromatography, followed by alternating low-and high-energy multiplexed MS/MS (UPLC–MSE) injections. Mass spectrometry analyses were further performed at a starting volume of 700 μL. Raw urine samples were then centrifuged at 10,000 × g for 30 min at 4°C, and the supernatants were perfused through a dialysis membrane with a 3,000 Da molecular weight cutoff (MWCO) (Amicon, Merck Millipore, Germany). The filtrate was desalted using a solid phase hydrophilic–lipophilic-balanced extraction cartridge (Oasis^®^ HLB, Waters Corporation, United States). The samples were concentrated using a SpeedVac Plus (Model: SC110A, ThermoSavant, United States) and reconstituted in solvent solution containing 3% acetonitrile and 0.1% formic acid in Milli-Q pure water. Finally, the samples were transferred to a UPLC autosampler vial (Waters Corporation, United States).

#### 2.2.1. UPLC–MSE method

UPLC–MSE data were acquired in an ultrahigh-performance liquid chromatography system (Acquity UPLC I-Class, Waters, United States) coupled to an ESI (+) Qq-oaTOF mass spectrometer (Xevo G2-S Q-Tof, Waters, United Kingdom). A total of 10 μL of each sample was injected, and the separation was performed on an ACQUITY UPLC CSH C18 column, 130 Å, 1.7 μm, 2.1 mm × 50 mm conditioned at 40°C. The mobile phases were 0.1% formic acid (pump A) and 0.1% formic acid in acetonitrile (pump B), and the flow rate was 900 μL min^−1^.

The gradient method was programmed to achieve maximum separation performance as follows: initial condition 3% B (pump B), 2.37 min 35% B, 4.37 min 85% B, 5.37 min 85% B, and 6.37 min 3% B with a total run time of 8.37 min and a calculated percent B/column volume (Cv) factor of 2.6%B/Cv. The sample tray temperature was defined at 8°C. The mass spectrometry method and conditions were adjusted, including alternating the continuous ion current with low-and high-energy multiplexed MS/MS mode (MSE) that could achieve a collision energy ramp set to 10^−30^ Ev.

MS was controlled by the MassLynx V4.1 software package (Waters Corporation, United Kingdom). The scanning mass range, quadrupole profile, and instrument calibration were set to transmit the ion current from *m*/*z* 50 to 1,000. The source conditions were tuned as follows: capillary at 3 kV, sampling cone (skimmer) set to 15 V, step wave source offset of 30 V, cone gas flow (curtain gas) set to 50 Lh^−1^, and desolvation temperature set to 550°C. All runs were acquired with a detector voltage of 2,450 V with the following hybrid analog-to-digital converter (ADC) parameters: an amplitude threshold of 2 V, an ion area threshold of 3 V, and an ion area offset of 15 V. Instrument calibration was achieved with an automatic Intellistart application included in the MassLynx software package (Waters, United Kingdom), and the acquisition was conducted with a solution of 0.1% formic acid:0.1 M NaOH:acetonitrile at a ratio of 1:1:8 to achieve less than 1 ppm across 14 monoisotopic masses, such as [M + H]+. The LockSpray setup was also performed prior to acquisition with leucine enkephalin (leu-enk) [M + H] + = 556.2771, and DRE lenses were automatically adjusted to allow for maximum transmission with a solution at 1 ng uL^−1^ and an infusion rate of 5 μLmin^−1^. An average ion area of 32 was also obtained from the detector setup with leu-enk.

#### 2.2.2. UPLC–MSE data processing with progenesis QI

Raw UPLC–MSE data files were processed and grouped by conditions as described previously (30). The identification and relative quantification based on ion accounting of putative metabolites were performed (default parameters) *via* Progenesis QI v.2.0 (Nonlinear Dynamics, Waters, United Kingdom). The metabolites were identified “on the fly” with the use of precursor ion exact mass, isotopologue distribution match, and fragment mass ion matching with the Human Metabolome Database (HMDB) and filtered with the urine metabolites database.

#### 2.2.3. Blood sampling and total carbon dioxide (TCO_2_) measurement

Accredited phlebotomists performed venipuncture, and samples of venous blood were taken from players (*n* = 27) at PRE and POST. Drops of blood were inserted into MetLyte 8 to measure TCO_2_ using a Piccolo Xpress (Abaxis, CA, United States).

### 2.3. Data analysis

Raw data are available in a previous report Prado et al. (7). Urine samples were normalized by specific gravity to ensure comparisons. Tyrosine metabolic pathways were investigated using the KEGG database. Data were filtered based on metabolite replication over individual analytical data acquisition, and data are presented as either up-, unchanged or down-regulated. After testing for normality (Shapiro–Wilk), the changes (PRE and POST) were analyzed using a paired Student’s *t*-test. Significance was set as *p* < 0.05.

## 3. Results

### 3.1. Soccer-induced hawkinsinuria

The homogentisate metabolites 4-maleylacetoacetate and succinylacetone were downregulated to 20 or 16% (i.e., 80 or 84% from the prematch), respectively, in response to the exercise protocol. 1,4-Benzoquinone acetate, a postulated homogentisate metabolite, also decreased to 10% of this original concentration. 4-Hydroxyphenylpyruvate, a precursor of homogentisate, was found to be upregulated by 26%. The concentration of hawkinsin and its metabolite 4-hydroxycyclohexyl acetate (4-HCCA) increased ~six-fold (Table 1). The 3-(4-hydroxyphenyl) lactate and 4-hydroxyphenylacetate metabolites of 4-hydroxyphenylpyruvate decreased to ~30 and ~60%, respectively (Table 1).

**Table 1:** Changes in metabolites related to tyrosine metabolism in the urine of athletes in response to a soccer match.

Compound	Relative concentration	Log (POST/PRE)	*t*-test (*p*)	HMDB ID
1,4-Benzoquinone acetate	DOWN	−1.0	3.00E−4	HMDB0002334
Carnosine	DOWN	−1.9	6.33E−13	HMDB0000033
DOPA	UP	0.6	5.62E−14	HMDB0000181
Dopamine 3-O-sulfate	DOWN	−1.2	2.21E−6	HMDB0006275
Dopaquinone	UP	0.8	4.98E−13	HMDB0001229
Glutamate	DOWN	−1.0	6.09E−9	HMDB0000148
Glutathione	DOWN	−0.4	2.17E−2	HMDB0000125
Hawkinsin	UP	0.8	1.49E−6	HMDB0002354
Homovanillin	UP	0.1	3.58E−2	HMDB0005175
4-Hydroxycyclohexyl acetate	UP	0.8	9.81E−6	HMDB0000451
4-Hydroxyphenylacetate	DOWN	−0.2	9.00E−4	HMDB0000020
3-(4-Hydroxyphenyl)lactate	DOWN	−0.5	4.55E−6	HMDB0000755
4-Hydroxyphenylpyruvate	UP	0.1	7.20E−3	HMDB0000707
Indole-5,6-quinone	DOWN	−1.0	9.66E−11	HMDB0006779
4-Maleylacetoacetate	DOWN	−0.7	4.69E−5	HMDB0002052
Melanin	DOWN	−2.1	4.81E−12	HMDB0004068
3-Methoxytyramine	UP	0.1	6.30E−3	HMDB0000022
3-Methoxytyrosine	DOWN	−0.6	1.75E−5	HMDB0001434
p-Octopamine	UP	0.1	5.24E−3	HMDB0004825
Pyroglutamate	UP	0.3	4.31E−2	HMDB0000267
Succinylacetone	DOWN	−0.8	4.25E−14	HMDB0000635
Tyramine O-sulfate	DOWN	−0.1	2.47E−2	HMDB0006409
Vanillactate	UNCHANGED	−0.1	6.12E−1	HMDB0000913

### 3.2. Tyrosine neurotransmitters changed pathways

The DOPA urinary concentration after the match increased four-fold from the pre-match, while dopaquinone increased by ~500% and 3-methoxytyramine increased ~30%. The sulfated forms of dopamine and tyramine, the predominant form of the compounds in blood, decreased to up to 5% or 80% compared to the pre-match concentrations. Other metabolites of DOPA significantly decreased in response to exercise. 3-methoxytyrosine, indole-5,6-quinone, and melanin were downregulated from 1% to 25% of PRE. Otherwise, homovanillin increased by 26% (Table 1).

### 3.3. Exercise changes In pH and REDOX

**Figure 1:**
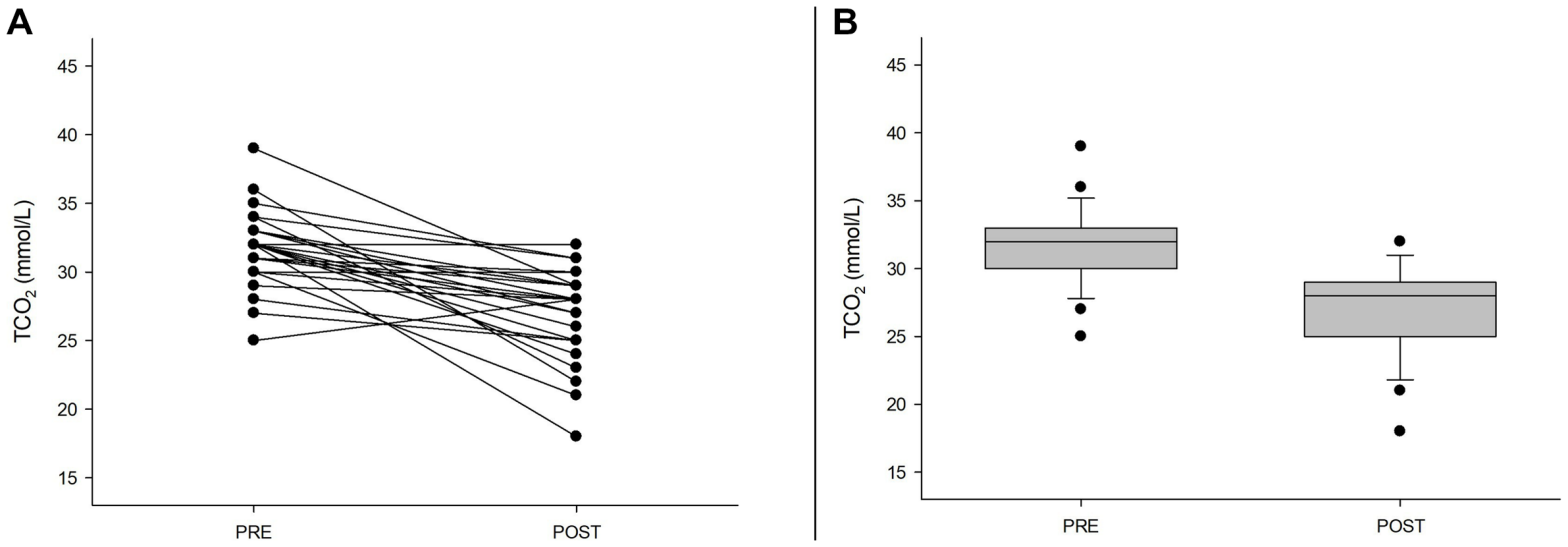
Exercise-induced decrease in blood TCO_2_. **(A)** Individual blood changes in TCO_2_ (*p* < 0.001). **(B)** Box plot showing the changes in TCO_2_ before (PRE) and after the match (POST).

Exercise is well known for decreasing pH and the buffering reserve inside muscle cells and blood. We measured a decrease in TCO_2_ as well as glutathione and glutamate (reaching 40% and 10% compared to pre-match) associated with a twofold increase in pyroglutamate in urine. Carnosine, a dipeptide with an essential role in pH maintenance, was downregulated to less than 2% compared to pre-exercise (Table 1 and Figure 1).

## 4. Discussion

We previously performed a sportomics investigation of soccer players during a match focused on purine metabolism Prado et al. (7). Here, some results were reexamined and reanalyzed on amino acid metabolism, leading us to tyrosine metabolism.

Mass spectrometry NTA can analyze a large number of metabolites present in human matrices (e.g., blood, urine, and saliva) and has potential applications in sports and exercise science (11). Urine is a convenient matrix due to its easy accessibility. Due to the systemic character of the urine, our data need to be interpreted as reflecting the sum of filtered blood metabolites.

Here, we show for the first time the upregulation of 4-hydroxyphenylpyruvate, hawkinsin, and 4-HCCA urinary excretion after exercise. We demonstrated an exercise-induced increase in hawkinsin in urine (which we propose to call exercise-induced hawkinsinuria; EIh). These unexpected findings are similar to those found in the inherited disorder Hawkinsinuria (ORPHA:2118) (12, 13). In this manuscript, we refer to Hawkinsinuria (with capital H) as the inherited disorder Hawkinsinuria (ORPHA:2118) and to hawkinsinuria as the presence of hawkinsin (2-amino-3-[2-(carboxymethyl)-2,5-dihydroxycyclohex-3-en-1-yl]sulfanylpropanoic acid) in urine. The physiopathology of Hawkinsinuria is not entirely understood, and the current knowledge regarding the role of hawkinsin in metabolism is limited to investigations of Hawkinsinuria. Patients with Hawkinsinuria can develop cognitive impairments, ataxia, and degradation in visual sensation, manifestations widely recognized as impairing sports performance (14).

Hawkinsinuria is caused by a mutation of the 4-hydroxyphenylpyruvate dioxygenase (4-HPPD, EC 1.13.11.27) gene, located in the chromosomal locus 12q24-qter, and is an autosomal dominant rare inborn error of metabolism characterized by acidosis and underdevelopment (15). It was previously described that the impairment of 4-HPPD leads to an increase in urinary hawkinsin in Hawkinsinuria (16). Our data revealed a significant increase in both hawkinsin and 4-HCCA in urine. The metabolism of 4-hydroxyphenylpyruvate remains to be elucidated and seems to be related to exercise (17). Under normal conditions, 4-HPPD catalyzes 4-hydroxyphenylpyruvate oxidation, decarboxylation, and final rearrangement to homogentisate. We measured a decrease in 3-(4-hydroxyphenyl)lactate and 4-hydroxyphenylacetate, together with a 10–20% urinary decrease in the metabolites succinylacetone, 4-maleylacetoacetate and 1,4-benzoquinone acetate as well as an increase in 4-hydroxyphenylpyruvate (~25%). These data may suggest an impairment of 4-HPPD activity in EIh. The decrease in 4-hydroxyphenylacetate was recently described in another soccer study (18).

In Hawkinsinuria, the 4-HPPD activity is impaired, hindering its rearrangement of an intermediate compound, generating a reactive epoxide. The epoxide can dissociate from 4-HPPD, ultimately producing quinoloacetate (19). Quinoloacetate can be drained to produce 4-HCCA or hawkinsin (20). In our study, we observed that a decrease in glutamate and glutathione is accompanied by an increase in pyroglutamate. Hawkinsinuria-related acidosis is not clearly understood, but it is believed to occur by pyroglutamate accumulation secondary to glutathione depletion (14). Children presenting Hawkinsinuria showed an increased urinary excretion of pyroglutamate during acidotic phases (20). Decreased glutathione levels can modify the y-glutamyl cycle, increasing the formation of pyroglutamate and saturating 5-oxoprolinase (EC 3.5.2.9).

The genesis of muscle H^+^ and subsequent blood acidosis seems to be related to massive ATP hydrolysis during muscle exercise (21). We measured different biomarkers, such as TCO_2_, glutathione, and carnosine, that are affected by a decrease in pH buffering reserve. Carnosine buffering may be used sooner in exercise to counteract acid production, being estimated to be approximately 7% of total muscle buffering (22–24). It is challenging to express causality during an intense exercise, such as a soccer match. In addition, carnosine deficiency may impair skeletal muscle metabolism (25).

In our study, the soccer match produced an upregulation of DOPA and dopaquinone concentrations in urine. The impact of exercise on tyrosine metabolism was briefly postulated (18). Both DOPA and tyramine metabolism seemed to be altered in our protocol. Tyrosinase is a busy enzyme in DOPA metabolism. The synthesis of indole-5,6-quinone and melanin seems to be impaired in our protocol, while DOPA and dopaquinone were up-regulated. Oxidation of DOPA and dopamine generates dopaquinone and dopaminoquinone, which are both neuron-cytotoxic molecules. Tyrosinase may rapidly oxidize excess amounts of cytosolic dopamine and DOPA in the brain, maintaining DOPA levels (26). The enzyme is present in a wide range of normal human organs, and some of its catalytic reactions seemed to be affected in our study (27). Additionally, in our study, the presence of tyramine metabolites was affected by the soccer match, suggesting possible changed routes for DOPA pathways in the central nervous system.

**Figure 2:**
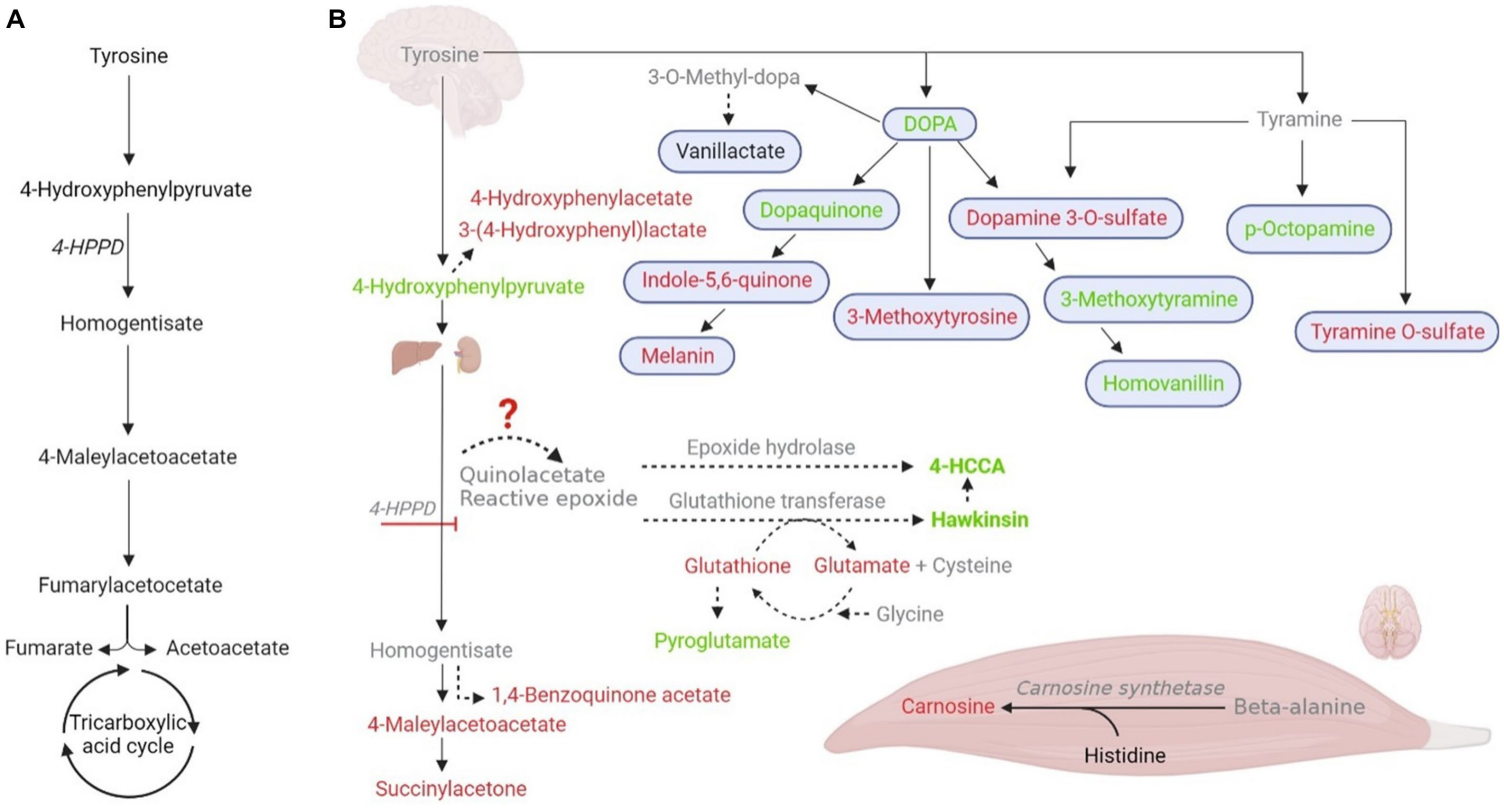
Postulated tyrosine metabolism changes during exercise, leading to an increase in hawkinsinuria. **(A)** Normal tyrosine metabolism and 4-hydroxyphenylpyruvate dioxygenase (4-HPPD; EC 1.13.11.27). **(B)** Metabolic measured changes in our study in response to exercise and their possible tissue specificity. Tyrosine metabolic-intermediate changes are shown in red (downregulation); green (upregulation); black (unchanged), and gray (not found). Solid arrows are established pathways, while dashed lines represent accessory pathways. Organs in the figure indicate the major metabolic contributors.

A 4-HPPD transient defect in newborns was already published (28). The transient defect in the newborn is probably caused by retarded maturation of 4-HPPD, together with impaired ascorbate ingestion associated with the high ingestion of protein, leading to an increase in hawkinsin formation (29). However, the impact of these findings on athletic performance must be further investigated to exploit the magnitude and reproducibility of these findings across sex, age, ethnic groups, and other important potentially modifying factors. We believe that EIh (which data we summarized in Figure 2) can be a transient condition of exercise metabolism, such as exercise-induced hyperammonemia (30), and exercise can be used as a model for the understanding of Hawkinsinuria and perhaps other tyrosine metabolism disorders.

## 5. Conclusion

The results suggest that under these conditions, exercise induces changes on amino acids metabolism, such as tyrosine metabolism. Combined, our results show that physical exercise can impair 4-HPPD activity, damaging the proper conversion of 4-hydroxyphenylpyruvate to homogentisate in normal tyrosine metabolism, which leads to hawkinsin urinary excretion. Therefore, the sportomics approach may be a suitable model for hawkinsinuria investigation. To the best of our knowledge, this is the first study to report physical exercise metabolism mimicking a rare genetic disorder. A future targeted replication and quantitative study will be necessary for a better understanding of the findings and showing how exercise stress could be used to study Hawkinsinuria and other metabolic disorders.

## Data availability statement

The original contributions presented in the study are included in the article/Supplementary material, further inquiries can be directed to the corresponding authors.

## Ethics statement

The studies involving human participants were reviewed and approved by Ethics Committee for Human Research at the Federal University of the State of Rio de Janeiro (117/2007, renewed in 2011). The patients/participants provided their written informed consent to participate in this study.

## Author contributions

MA, ABo, ABa, and LC: conceptualization. TF and RM-S: visualization. AS and LC: funding acquisition. EP and LC: supervision. TF, RM-S, EP, and LC: writing—original draft and writing—review and editing. All authors contributed to the article and approved the submitted version.

## Funding

This study was supported and funded by Conselho Nacional de Desenvolvimento Científico e Tecnológico (CNPq), Coordenação de Aperfeiçoamento de Pessoal de Nível Superior (CAPES), Financiadora de Estudos e Projetos, Fundação Carlos Chagas Filho de Amparo à Pesquisa do Estado do Rio de Janeiro, Merck-Sigma-Aldrich, Universidade Federal do Estado do Rio de Janeiro and Waters Corporation.

## Conflict of interest

GS was employed by Former Waters Corporation, Currently at SpectraMass.

The remaining authors declare that the research was conducted in the absence of any commercial or financial relationships that could be construed as a potential conflict of interest.

## Publisher’s note

All claims expressed in this article are solely those of the authors and do not necessarily represent those of their affiliated organizations, or those of the publisher, the editors and the reviewers. Any product that may be evaluated in this article, or claim that may be made by its manufacturer, is not guaranteed or endorsed by the publisher.
